# Sustained hyperuricemia disrupts enterohepatic circulation through 12α-hydroxy BA and gut microbiota

**DOI:** 10.3389/fmicb.2025.1671409

**Published:** 2025-11-10

**Authors:** Tong Zou, Ruixiao Zhang, Zeqing Chen, Haiping Duan, Zhaoguo Wang, Ruipeng Gao, Xin Chen, Hongqiang Wang, Yue Zhao, Xiaofeng Wang, Shichao Xing

**Affiliations:** 1 Women and Children’s Hospital, Qingdao University, Qingdao, China; 2 Department of Emergency, The Qingdao Municipal Hospital, Dalian Medical University, Dalian, China; 3 The Qingdao Municipal Hospital, University of Health and Rehabilitation Sciences, Qingdao, China; 4 Qingdao Municipal Center for Disease Control and Prevention, Qingdao, China; 5 Affiliated Hospital of Qingdao University, Qingdao University, Qingdao, China; 6 School of Cardiovascular Medicine and Science, King’s College London, BHF Centre, London, United Kingdom

**Keywords:** hyperuricemia, gut microbiota, bile acid, 12α-hydroxy, uric acid

## Abstract

Hyperuricemia (HUA) has become a worldwide health issue, drawing increasing public attention. This study aimed to elucidate the roles of bile acids (BAs) and their associations with gut microbiota in a HUA rat model. We established normal control (N) and HUA rat groups, then characterized the BA profiles in liver, serum, and ileum contents using a targeted metabolomics approach. Additionally, gut microbiota composition was analyzed through 16S rDNA gene sequencing. The results were that hyperuricemia induced elevated levels of cholic acid (CA) in liver and elevated levels of taurocholic acid (TCA), glycocholic acid (GCA), and taurodeoxycholic acid (TDCA) in the ileum content, particularly the increases in the levels of total 12α-hydroxy bile acid in the ileum content, which were consistent with the increased levels of CA in the liver. These changes are correlated to an increase in the abundance of the genera *Allobaculum* and *Bifidobacterium*. Our investigation revealed the fluxes of bile acids and their association with gut microbiota in HUA and provides new ideas for the study of metabolic diseases in hyperuricemia.

## 1 Introduction

Hyperuricemia (HUA), defined as elevated serum uric acid (UA) levels, has been linked to obesity, fatty liver disease, and type 2 diabetes (Wan et al., 2016; Civantos Modino et al., 2012; Kodama et al., 2009). In some developed countries, approximately 20.2% of men and 20.0% of women are affected by HUA (Chen-Xu et al., 2019). Furthermore, studies have highlighted a contributory role of UA in metabolic syndrome, a condition characterized by lipid metabolism disorders and insulin resistance (Zavaroni et al., 1993; Feng et al., 2022; Meng et al., 2022). Due to its widespread impact, HUA has become a significant worldwide health issue and attracted considerable public attention.

Bile acids assist in fat absorption in the intestine and play key roles in regulating lipid and glucose metabolism. Synthesis of BAs is regulated by three cholesterol hydroxylase enzymes, mitochondrial sterol 27-hydroxylase (CYP27A1), cholesterol 7α-hydroxylase (CYP7A1), and sterol 12α-hydroxylase (CYP8B1) (Liu et al., 2020). CYP7A1 and CYP27A1 are the rate-limiting enzymes of classical and alternative pathways, respectively (Thomas et al., 2008). The classical pathway synthesizes chenodeoxycholic acid (CDCA) and cholic acid (CA), while the alternative pathway synthesizes CDCA. The ratio of CDCA/CA is determined by CYP8B1 (Li-Hawkins et al., 2002), which is a BA 12α-hydroxylase that determines the production of CA (Bertaggia et al., 2017). CA can be further modified by intestinal microbes into secondary BAs. In previous studies, it has been demonstrated that 12α-hydroxy BAs are highly positively correlated with fat levels, and 12α-hydroxy BAs are closely related to the pathogenesis of lipid metabolism disorders (Iwasaki et al., 2022).

Gut microbiota converts primary bile acid to secondary bile acid in hepatic-intestinal circulation. Many studies have found gut microbiota alterations in HUA (Wei et al., 2022; Yu et al., 2018), which may induce the altered BA composition. Previous studies have shown that the gut microbiota is not only involved in converting BAs but also in regulating the biosynthesis of several bile acid synthesis enzymes, including CYP7A1 and CYP27A1(Sayin et al., 2013; Ridlon et al., 2006). Some research has reported that the development of metabolic disorders was accompanied by the alteration of gut microbiome and metabolites, particularly bile acids (Lin et al., 2019; Jiao et al., 2018).

In the present research, we aimed to investigate the impact of HUA changes in a rat model with emphasis on the interplay between bile acids and gut microbiota. To the best of our knowledge, it is the first study that focused on the investigation of bile acids in the enteric-hepatic cycle and their interplay with the microbiome during the development of HUA.

## 2 Materials and methods

### 2.1 Animal study

The animal experiments were approved by Qingdao University (Qingdao, China). All animal experiments followed the guide for the care and use of laboratory animals. Female Wistar rats (8 weeks old) were purchased from Vital River Laboratory Animal Technology Co., Ltd. (Beijing, China). All rats were raised as four or three per cage in clear plastic cages in a specific pathogen-free temperature-controlled environment with a 12-h light/dark cycle (lights on from 8:00 a.m. to 8:00 p.m.) and ad libitum access to water and food. All animals were acclimatized for 1 week before the experiment. Female rats were randomly divided into two groups.

The HUA group was gavaged with adenine at 100 mg/(Kg·d), and, after 5 weeks, the dose of adenine was reduced to 50 mg/(Kg·d), while at the same time, HUA (n = 7) rats were intraperitoneally injected with potassium oxonate (PO) at 50 mg/(Kg·d). The normal (n = 7) group was gavaged with the same amount of sterile water. After 9 weeks, fresh feces were obtained by stimulating the anus. Rats that had been fasted for 6 h were anesthetized with isoflurane, and the blood samples were collected from the intra-orbital retrobulbar plexus. Livers, ileum contents, and serum were stored at −80 °C until assayed.

### 2.2 Bile acid analysis

Bile acid analysis was conducted at Lipid ALL Technologies. In brief, bile acids were extracted from serum using ice-cold methanol: acetonitrile (5:3) containing deuterated internal standards including glycocholic acid-d4, glycochenodeoxycholic acid-d9, glycodeoxycholic acid-d4, cholic acid-d4, ursodeoxycholic acid-d4, chenodeoxycholic acid-d4, deoxycholic acid-d4, and lithocholic acid-d4 (Avanti Polar Lipids) as previously described. Clean supernatant was transferred to a new tube and dried in the SpeedVac™ vacuum concentrator (Thermo Fisher Scientific, USA) under the OH mode. The extract was resuspended in 50 μL of methanol and analyzed on an Exion AD30-UPLC (Sciex, USA) coupled with a Sciex QTRAP 6500 Plus (Sciex, USA) under the electrospray ionization mode. Individual bile acids were separated on a Phenomenex Kinetex C18 column (100×2.1 mm, 1.7 μm) using 2% formic acid in water as mobile phase A and acetonitrile:isopropanol (1:1) as mobile phase B and quantitated by referencing the intensities of their corresponding deuterated internal standards.

Tissues were homogenized in 40% methanol on a bead ruptor (OMNI, USA). The samples were incubated at 1,500 rpm for 30 min at 4 °C. The samples were centrifuged at the end of incubation, and the clean supernatant was extracted. The extraction was repeated with ice-cold methanol:chloroform (3:1), and the extracts were pooled. The pooled extracts were dried in a SpeedVac vacuum concentrator under the OH mode and resuspended in 50 μL methanol containing deuterated internal standards prior to LC–MS analysis. The internal standard cocktail contained glycocholic acid-d4, glycochenodeoxycholic acid-d9, glycodeoxycholic acid-d4, cholic acid-d4, ursodeoxycholic acid-d4, chenodeoxycholic acid-d4, deoxycholic acid-d4, and lithocholic acid-d4 (Avanti Polar Lipids). Bile acids were analyzed on an Exion AD30-UPLC coupled with Sciex QTRAP 6500 Plus under the electrospray ionization mode of negative polarity. Individual bile acids were separated on a Phenomenex Kinetex C18 column (100×2.1 mm, 1.7 μm) using 2% formic acid in water as mobile phase A and acetonitrile: isopropanol (1:1) as mobile phase B and quantitated by referencing he intensities of their corresponding deuterated internal standards.

The levels of 12α-OH BAs were the sum of the concentration of CA, deoxycholic acid (DCA), taurocholic acid (TCA), glycocholic acid (GCA), taurodeoxycholic acid (TDCA), and glycodeoxycholic acid (GDCA) in each sample (Xie et al., 2021).

The HPLC–MS analysis was performed according to proprietary protocol.

Methanol (HPLC grade; Fisher Chemical, A452-4), acetonitrile (LC–MS grade; Fisher Chemical, A955-4), isopropanol (99.9%; Fisher Chemical, A451-4F), chloroform (HPLC grade; Honeywell, 049-4), and formic acid (98%; J&K 299272) were used in this study. Solvents were purchased from Sigma-Aldrich (USA) or the suppliers and catalogue numbers given above.

### 2.3 16S rDNA sequencing

Microbial community genomic DNA was extracted from fecal samples using the E.Z.N.A.^®^ soil DNA Kit (Omega Bio-tek, Norcross, GA, USA). 16s rRNA genes (V3–V4 variable regions) were amplified with the primers 338F (50-ACTCCTACGGGAGGCAGCAG-30) and 806R (50-GGACTACHVGGGTWT CTAAT-30). The amplification reaction was performed in a 25 μL volume containing 12.5 μL of Phusion Hot Start Flex 2X Master Mix, 2.5 μL of forward primer, 2.5 μL of reverse primer, and 50 ng of template DNA. The reaction was performed with the following polymerase chain reaction (PCR) program: denaturation at 98 °C for 30 s, followed by 35 cycles of denaturation at 98 °C for 10 s, annealing at 54 °C for 30 s, extension at 72 °C for 45 s, and a final extension at 72 °C for 10 min. The PCR product was purified through agarose gel (2%) electrophoresis, and commercial sequencing was conducted on the Illumina MiSeq platform.

### 2.4 Real-time quantitative polymerase chain reaction

Total RNA from the tissues was extracted using Trizol reagent (Takara, People’s Republic of China). The total RNA was used to obtain cDNA using All-In-One 5X RT MasterMix (No. G592, ABM, People’s Republic of China). The obtained cDNA was used for a real-time PCR reaction with BlasTaq qPCR 2X MasterMix (No. G891, G892, ABM, China). The primers used are shown in Table 1.

**Table 1:** Rat primers used for research.

Gene	Forward (5′ → 3′)	Reverse (5′ → 3′)
GAPDH	GGCACAGTCAAGGCTGAGAATG	ATGGTGGTGAAGACGCCAGTA
CYP7A1	AGGTCTCTGAACTGATCCGTCTACG	GAGAATAGCGAGGTGCGTCTTGG
CYP27A1	TCGCACCAATGTGAATCTGGCTAG	CCACTGCTCCATGCTGTCTCTTATG
CYP8B1	GTCAGGCAAGAAGATCCACCACTAC	GTCAGGGTCCACCAGTTCAAAGTC

### 2.5 Western blot analysis

The liver and ileum tissues were homogenized and centrifuged at 13,000*g* at 4 °C for 10 min. The supernatant was a protein sample. After the sample was denatured and boiled, the extracted proteins were separated using sodium dodecyl sulfate–polyacrylamide gel electrophoresis (SDS-PAGE) and then transferred to a polyvinylidene fluoride (PVDF) membrane (Millipore). After blocking with 5% milk for 90 min, the membrane was washed three times with phosphate-buffered saline (PBS) containing Tween 20 (PBST) and incubated overnight with the indicated primary antibodies at 4 °C. The antibodies used were as follows: rabbit anti-*β*-actin (D110001-0025, 1:1,000, Sangon Biotech), rabbit anti-CYP7A1 (Cat No. F2612, 1:500, Affinity), and rabbit anti-CYP8B1 (abs145876, 1:500, absin). The next day, the membrane was washed with PBST and incubated with horseradish peroxidase-conjugated secondary antibodies at room temperature (RT) for 1 h. Anti-rabbit IgG-linked and HRP-linked antibodies (abs20040, 1:5,000, absin) were used. The protein contents were detected using an imaging system and a chemiluminescence kit (Millipore, MA, USA) and quantitated using ImageJ software (Bio-Rad) to analyze the gray value of the blots. Protein expression was normalized to that of β-actin.

### 2.6 Statistical analysis

The results are expressed as mean ± SD. Statistical significance was analyzed using the unpaired Student’s t-test. The Mann–Whitney U test was used for BA data analysis because the majority of the data were not normally distributed. The software package GraphPad Prism (GraphPad Software 8.0, La Jolla, CA, USA) was used to statistically analyze the results. A *p*-value of < 0.05 was considered to be statistically significant. Principal component analysis (PCA) was carried out in the R programming environment (version 4.2.1), using the factoextra package (version 1.0.7) for visualization.

## 3 Results and discussion

### 3.1 Persistent increase of uric acid concentration in HUA rats

**Figure 1:**
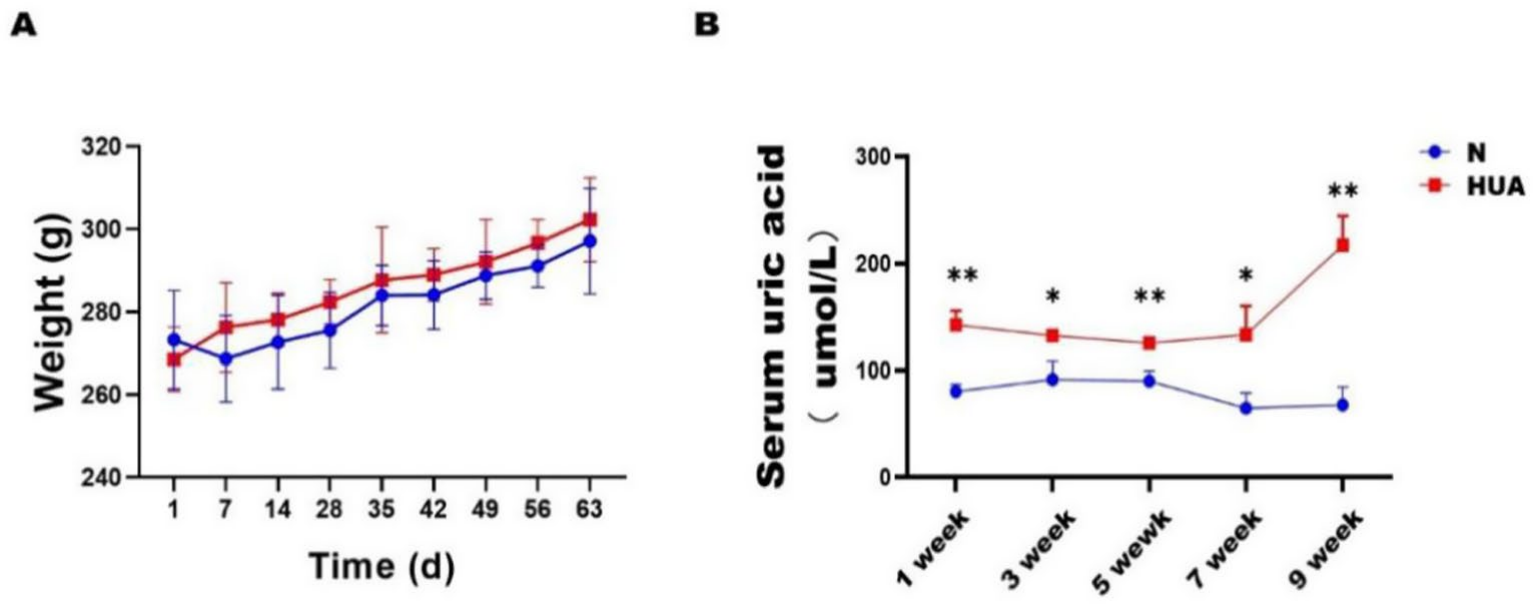
Lipid metabolism disorders in HUA rat. **(A)** Body weight. **(B)** Serum UA. Data are shown as means ± SDs (*n* = 3). *p < 0.05, **p < 0.01, ***p < 0.001 (unpaired Student’s t-test). HUA, hyperuricemia; N, normal.

There was no significant difference in body weight between the HUA and normal (N) groups (Figure 1A). The UA content in the HUA group was significantly higher than that in the N group from the 1st to the 9th week (Figure 1B).

### 3.2 HUA-induced changes in the ileum 12α-hydroxy bile acids

**Figure 2:**
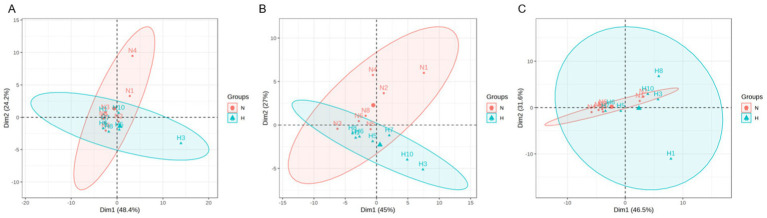
Dysregulated bile acid principal component in the HUA rats. **(A–C)** Principal component analysis (PCA) score plot of serum **(A)**, liver **(B)**, and ileum contents. **(C)** Bile acid profiles. Principal component analysis (PCA) of groups N and H. The separation between the groups was visualized in the PCA plots and further validated by the PERMANOVA (Adonis) test based on the Bray–Curtis/Euclidean distance matrices, which confirmed significant differences between the groups (*p* < 0.05).

BAs play an important role in lipid metabolism as signaling molecules. To assess the BA profile in the HUA and N groups of rats, liquid chromatography–mass spectrometry (LC–MS) was applied to acquire their BA profiles. To evaluate whether the separation observed in PCA was statistically significant, we performed a permutational multivariate analysis of variance (PERMANOVA) (Adonis) test based on the Bray–Curtis/Euclidean distance matrices, using the vegan package in R. The test was conducted with 999 permutations, and a p-value < 0.05 was considered statistically significant. We observed a clear separation between the N group and HUA group rats from the principal component analysis (PCA) model established with the identified liver (Figure 2B) and ileum (Figure 2C) bile acids; there was no separation between the N and HUA groups in serum bile acids (Figure 2A).

### 3.3 HUA-induced changes in serum, liver, and ileum bile acids

**Figure 3:**
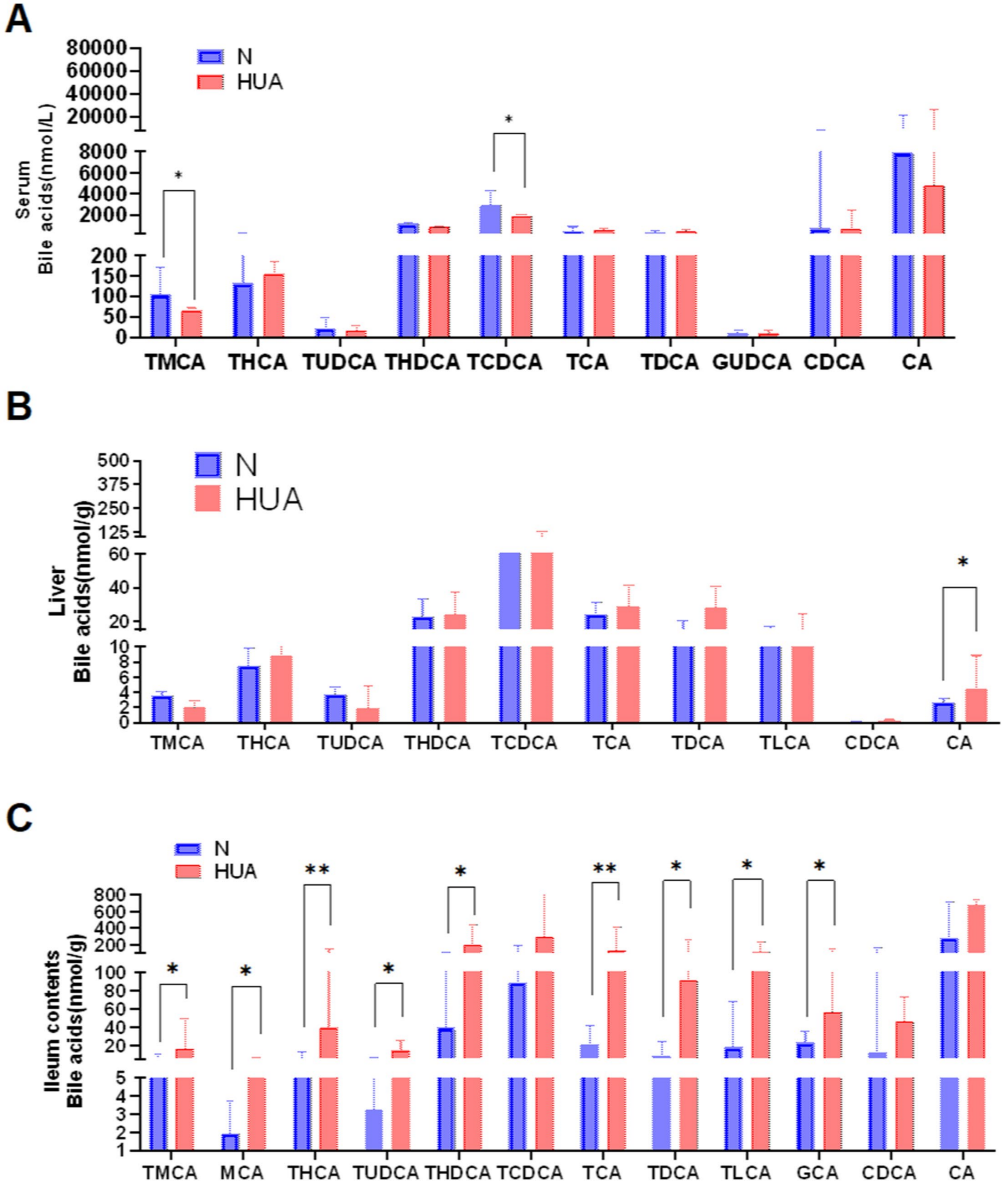
Dysregulated bile acid profile in HUA rats. **(A–C)** Individual BAs concentrations of serum **(A)**, liver **(B)**, and ileum contents **(C)**. Data are shown as median (Q1, Q3) (n = 4–5, Mann–Whitney U test). ^*^p < 0.05, ^**^p < 0.01.

To explore the dynamic changes of BAs, we collected the serum, liver, and ileum contents of N and HUA group rats. In serum, we found that taurochenodeoxybile acid (TCDCA) and tauro-muribile acid (TMCA) decreased significantly compared with the N group (Figure 3A). In the liver, we found that CA increased dramatically compared with the N group (Figure 3B). More importantly, in ileum contents, we found that TMCA, muricholic acid (MCA), taurohyocholic acid (THC), tauroursodeoxycholic acid (TUDCA), taurohyodeoxycholic acid (THDCA), glycoursodeoxycholic acid (GUDCA), TCA, taurodeoxycholic acid (TDCA), taurolithocholic acid (TLCA), and GCA increased remarkably (Figure 3C).

### 3.4 HUA-induced changes in the ileum 12α-hydroxy bile acids

**Figure 4:**
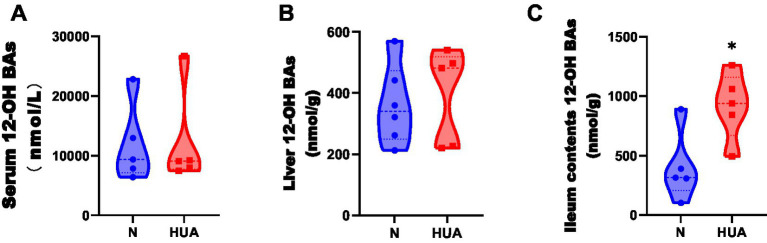
Dysregulated 12α-hydroxy bile acid concentrations in HUA rats. **(A–C)** 12α-hydroxy BA concentrations of serum **(A)**, liver **(B)**, and ileum contents **(C)**. Data are shown as median (Q1, Q3) (n = 5–6, Mann–Whitney U test). ^*^p < 0.05, ^**^p < 0.01.

There was no change in 12α-hydroxy BAs in the liver, but 12α-hydroxy BAs significantly increased in ileum contents compared with the N group (Figures 4A–C).

### 3.5 HUA-induced changes in CYP7A1 and CYP8B1

**Figure 5:**
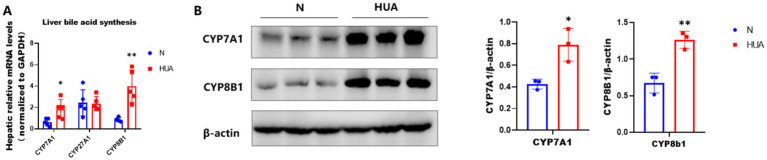
Increased expression of CYP7A1 and CYP8B1 in HUA. **(A)** CYP7A1, CYP27A1, and CYP8B1 gene mRNA abundances. **(B)** CYP7A1 and CYP8B1 protein contents. Data are shown as means ± SDs (n = 5; unpaired Student’s t-test). ^*^p < 0.05, ^**^p < 0.01.

To determine the cause of the increase in 12α-hydroxy bile acids in the ileum, we observed the bile acid synthesis pathway. We found that rats with HUA exhibited significant upregulation of hepatic mRNA abundances of CYP7A1 and CYP8B1 genes, instead of CYP27A1 genes, and increased contents of CYP7A1 and CYP8B1 (Figures 5A,B).

### 3.6 HUA-induced changes in gut microbiome

**Figure 6:**
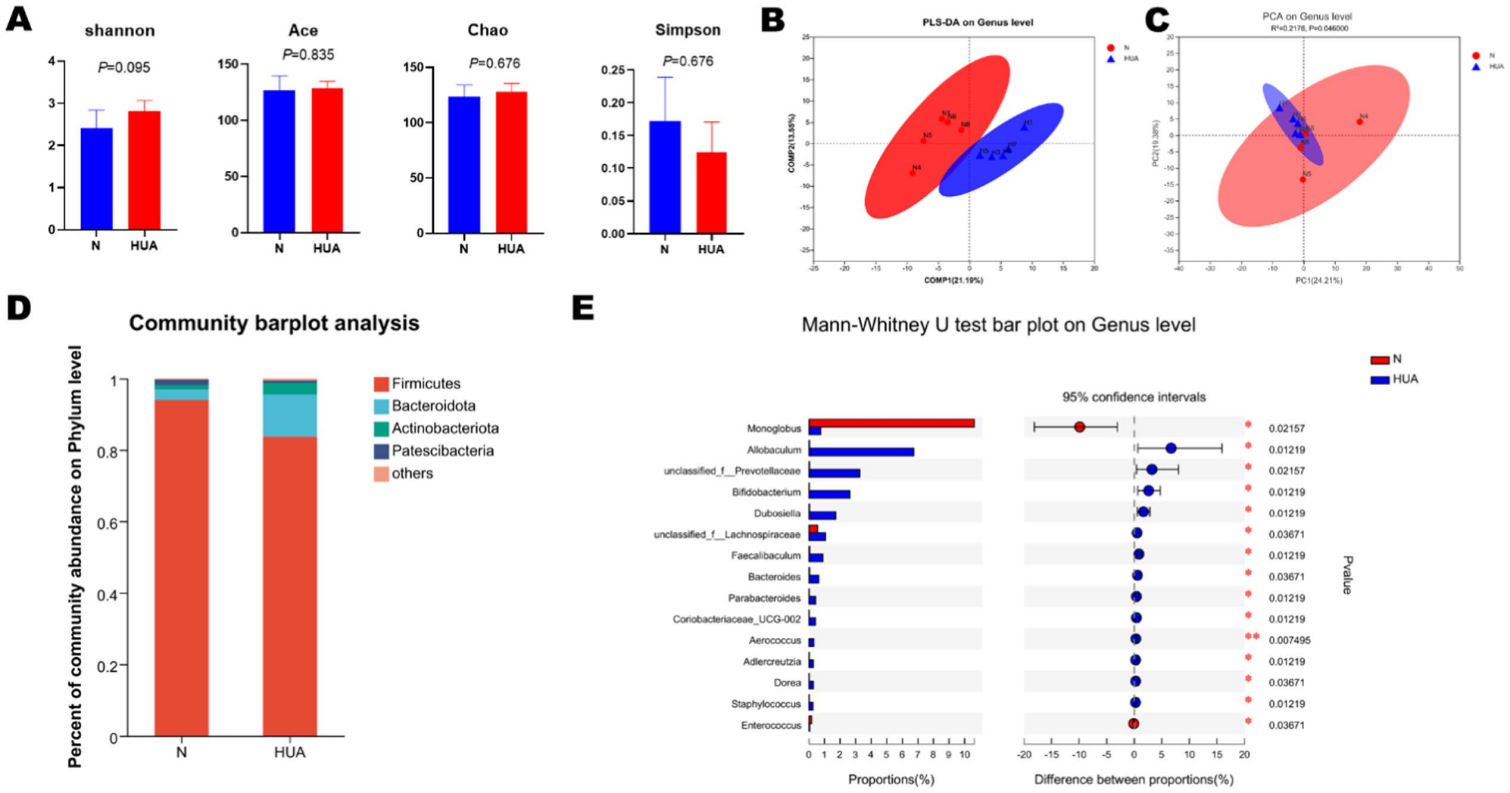
Altered BA profiles and their association with gut microbiota. **(A)** α-diversity of gut microbiota, including Shannon, Simpson, Ace, and Chao indices. Data are shown as means ± SDs (n = 5, Mann–Whitney U test). **(B,C)** The principal component analysis **(B)** and partial least-squares discriminant analysis **(C)** at the genus level identified by metagenomic sequencing (n = 5). **(D)** Relative proportion of the phylum in each group. **(E)** Relative abundance of the top 15 genera in each group. Data are shown as median (Q1, Q3) (n = 5, Mann–Whitney U test).

The rat fecal microbiota composition was detected by sequencing the respective 16S rDNA genes, and the gut microbiota of five rats in each group was analyzed. The Shannon, Simpson, Ace, and Chao indices were found unchanged (Figure 6A). At the genus level, we found that there was obvious separation between the groups in microbiota (Figure 6B). The bacterial communities in the HUA group and the matched controls clustered separately, suggesting that there was a remarkable difference between the N and HUA groups (Figure 6C). At the phylum level, the proportion of Bacteroidetes increased in the HUA group, and the proportion of Firmicutes decreased compared to the control (Figure 6D). There was a remarkable difference in bacterial communities among the groups. *Allobaculum*, *unclassified_f__Prevotellaceae*, *Bifidobacterium*, *Dubosiella*, *unclassified_f__Lachnospiraceae*, *Faecalibaculum*, *Bacteroides*, *Parabacteroides*, *Coriobacteriaceae_UCG-002*, *Aerococcus*, *Adlercreutzia*, *Dorea,* and *Staphylococcus* increased, while *Monoglobus* and *Enterococcus* decreased compared with the N group (Figure 6E).

### 3.7 The correlation between 12α-hydroxy bile acids and gut microbiome in HUA rats

**Figure 7:**
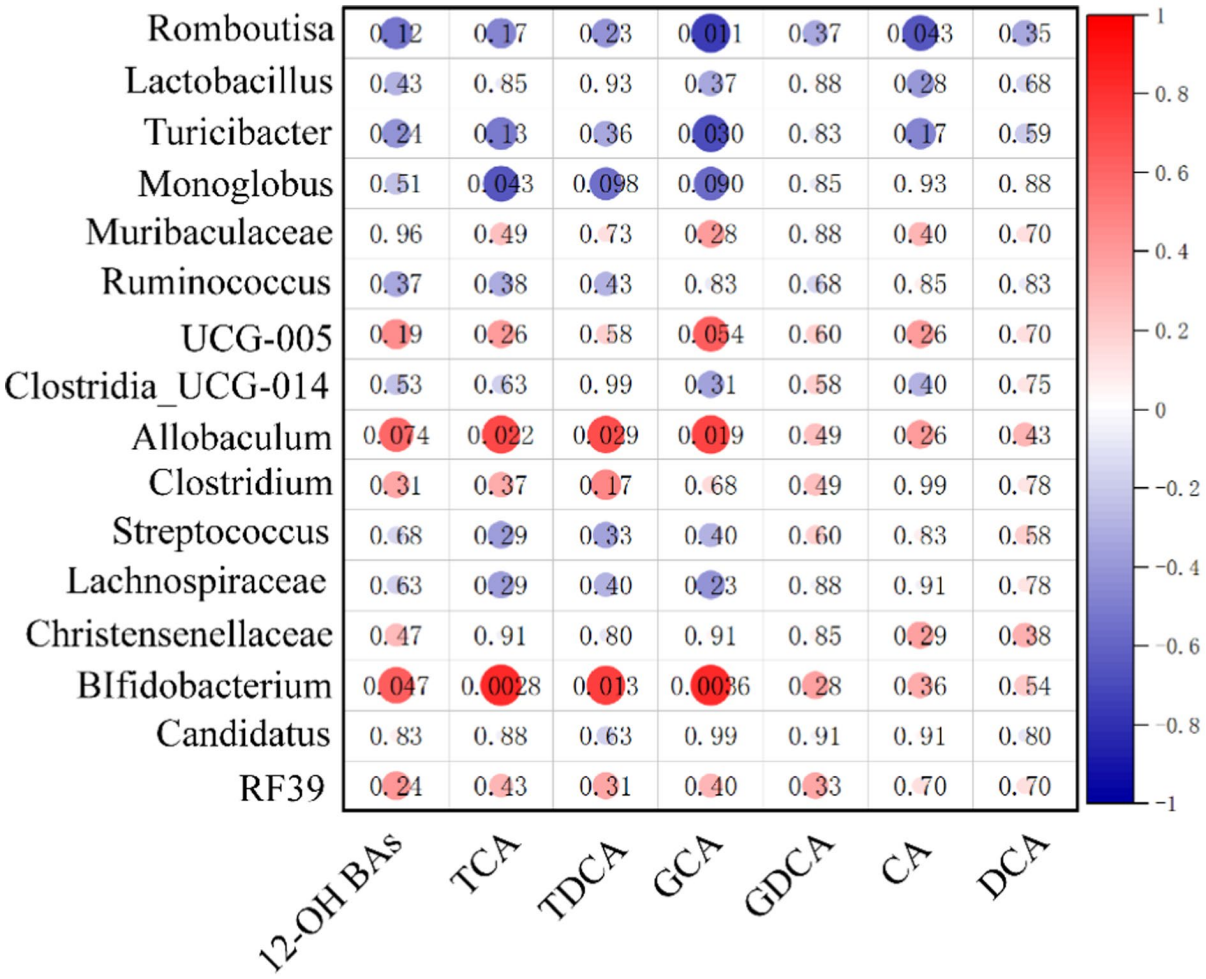
Heatmap of Spearman correlation coefficients between ileum contents 12α-hydroxy and microbiota. The gradient colors represent the correlation coefficients, with red being more positive and blue being more negative (n = 10, 5 samples per group). ^*^p < 0.05, ^**^p < 0.01.

To visualize the correlation between the gut microbiota and BA abundances, the Spearman correlation was conducted between the main 12α-hydroxy BA abundances in ileum contents and the relative abundances of the differential bacterial genus identified above. We found that *Allobaculum* and *Bifidobacterium* had significantly positive correlations with total 12α-hydroxy BA, TCA, TDCA, and GCA. These results suggest that gut microbiota changes may impose a substantial impact on the 12α-hydroxy BA composition (see Figure 7).

## 4 Discussion

Uric acid is widely regarded as the final metabolic product of purines in humans (McQueen et al., 2012). Unlike humans, uricase expressed in the liver of rodents can further degrade UA into allantoin (Álvarez-Lario and Macarrón-Vicente, 2010), which has hindered the establishment of suitable rodent models for HUA. In this study, we used adenine to simulate the purine intake in the daily human diet, and uricase was inhibited with PO. High-dose adenine can cause nephrotoxicity, significantly increasing the mortality rate of animals, which is not conducive to the stable establishment of long-term models. Therefore, we drew on the experience of previous model establishment (Liu et al., 2020), first increasing the uric acid level with high-dose adenine and then reducing the adenine dose in the 5th week to maintain a high level of blood uric acid while significantly reducing its severe nephrotoxicity and establishing a stable chronic model of hyperuricemia. Through continuous monitoring of uric acid levels, the uric acid level in the HUA group remained consistently higher than that in the control group and reached its peak at 7–9 weeks. This might be due to potassium oxazinate inhibiting the continuous accumulation and saturation of uric acid decomposition in the body, which completely disrupts the balance between uric acid production and catabolism, thereby causing a sudden increase in SUA levels. This phenomenon also indicates that the model has been successfully established.

The ratio between CDCA and CA is determined by the sterol 12a-hydroxylase (CYP8B1), which is required for CA synthesis. CYP8B1 is a BA 12α-hydroxylase that determines the production of cholic acid (CA, which is 12α-hydroxylated). CA can be further modified by intestinal microbes into secondary BAs, such as DCA, TCA, TDCA, GCA, and GDCA, which are generated from CA and are 12α-hydroxylated. In our study, the expression of cytochrome P7A1 and cytochrome P8B1 induced by HUA was generally increased, while the gene expression of CYP27A1, the initiating enzyme of the alternative pathway, remained unchanged. It should be noted that the alternative pathway also involves another key enzyme, namely oxysterol 7α-hydroxylase (CYP7B1), which acts downstream of CYP27A1 to promote the synthesis of chenodeoxycholic acid (CDCA) (Wahlström et al., 2016). Although the expression of CYP7B1 was not detected in this study, the fact that the expression of the CYP27A1 gene did not change may suggest that under our experimental conditions, the alternative pathway may not be mainly activated. However, we cannot rule out the possibility that post-transcriptional regulatory or compensation mechanisms affect CYP7B1 activity. Future research, by specifically detecting the expression and activity of CYP7B1, will be of great significance for comprehensively clarifying the role of alternative pathways in the observed bile acid profile disorder in hyperuricemia.

We also discovered the elevated CA level in HUA rat liver and the significantly elevated TCA, TDCA, GCA, and total 12α-hydroxy BA levels in HUA rat ileum contents. In the ileum of obese mice, CA and TMCA levels were increased (Duan et al., 2019), and patients with NAFLD exhibited increased CA levels (Jiao et al., 2018). Our results are consistent with these studies. It has been demonstrated that 12α-hydroxy BAs aggravate body weight, liver steatosis, and lipid homeostasis. 12α-hydroxy BAs are the therapeutic target for obesity, fatty liver, and hypertriglyceridemia (Hori et al., 2020). Therefore, we believe that 12α-hydroxy BAs are closely related to the development of lipid metabolism disorders in HUA.

In this study, our data show decreased phyla *Firmicutes* and increased *Bacteroidota* in HUA. Previous studies did not clarify the changes in *P*. *Firmicutes* and *P*. *Bacteroidota* in HUA. In addition, some studies have found a significant decrease in *P*. *Firmicutes* and a significant increase in *P*. *Bacteroidota* in HUA (Yu et al., 2018; Cao et al., 2022; Sun et al., 2022), while some others and our previous studies have found the opposite result (Liu et al., 2020; Pan et al., 2020). The abundance of phyla *Firmicutes* and *Bacteroidota* in HUA with and without symptoms is different (Kim et al., 2022). Similar to HUA, there is a lack of definitive findings on the alterations of phyla *Bacteroidota* and *Firmicutes* in NAFLD (Aron-Wisnewsky et al., 2020). Gut microbiota analysis revealed that the phyla *Bacteroidota* and *Firmicutes* had high bile salt hydrolase (BSH) activity (Jones et al., 2008). We therefore speculate that changes in the phyla *Bacteroidota* and *Firmicutes* may lead to changes in BSH activity in HUA. At the genus level, the abundance of *Prevotella*, *Bifidobacterium*, *Bacteroides*, *Parabacteroides,* and *Dorea* was found to increase in NAFLD (Aron-Wisnewsky et al., 2020), and *Allobaculum* was found to increase in HFD mice (Zheng et al., 2021). Our results are consistent with the above-mentioned results that the abundance of *Prevotella, Bifidobacterium, Bacteroides, Parabacteroides,* and *Dorea* increased in HUA groups. It is worth noting that, in addition to BSH activity, the transformation of bile acids is deeply influenced by the bile acid-induced (bai) operon, which is responsible for generating secondary bile acids through 7α-dehydroxylation—a process mainly dominated by certain *Clostridium* and *Bacteroides* species (Meibom et al., 2024; Ridlon and Hylemon, 2012). These biochemical modifications significantly alter the hydrophobicity, signal transduction characteristics, and physiological effects of bile acids, ultimately influencing host metabolism. Although the bai gene activity was not directly measured in this study, the changes in the composition of the intestinal microbiota in HUA rats suggest that the metabolism mediated by the bai gene may have changed, which may have promoted the disorder of the overall bile acid pool and is worthy of further research. We found evidence of dysbiosis in the HUA rat gut microbiota, and these changes may be partially consistent with changes in obesity and NAFLD.

*Allobaculum* is considered harmful to improve lipid metabolism in the genus. *Allobaculum* abundance was significantly higher in aged obese mice (Kain et al., 2019), and similarly in NAFLD rats, the abundance of *Allobaculum* was significantly and positively correlated with triglycerides and cholesterol (Tang et al., 2018). Recent evidence suggests that *Bifidobacterium* can conjugate, rather than only deconjugate, including conjugates of CA, DCA, and CDCA (Tsutsui et al., 2011; Lucas et al., 2021). Conjugated bile acids have greater water solubility and are secreted into bile; meanwhile, conjugated bile acids can be more easily taken up by ileal bile acid transporters/apical bile acid transporters (IBAT/ABAT) (Begley et al., 2006). In our investigation, the *Allobaculum* and *Bifidobacterium* had significantly positive correlations with total 12*α*-hydroxy BA, TCA, TDCA, and GCA. Among these two bacteria, *Bifidobacterium* is known to produce CA into conjugated bile acids, which may make it easier for 12α-hydroxy BAs to remain in the enterohepatic circulation rather than being excreted in the feces.

Although this study revealed the changes in bile acid metabolism and gut microbiota under hyperuricemia and their potential association, there are still some limitations: This study mainly revealed the changes in the correlation between bile acid profiles and gut microbiota under the HUA state. We have not yet directly confirmed through functional experiments that the identified specific bacterial communities (such as *Allobaculum* and *Bifidobacterium*) are the cause of the increase in 12α-hydroxy bile acid, rather than the result. Further studies, such as fecal microbial transplantation or antibiotic clearance experiments, will be conducted to establish the causal relationship between microbial changes and bile acid metabolism in hyperuricemia. This study only used rat models, and the sample type was single. In the future, we can analyze the serum and fecal bile acid profiles of patients with hyperuricemia or gout, as well as the structure of their intestinal flora, to confirm whether the elevated ratio of 12α-hydroxy to non-12α-hydroxy bile acids and the association with specific flora also exist in humans. This will provide clinical evidence for the development of novel microecological preparations or dietary intervention strategies targeting the gut microbiota-bile acid axis; Due to sample loss during the sample processing, the reduced sample size may affect the statistical power and universality of our research results. In the future, we will use a larger sample size to further verify and expand these preliminary observations. Female rats were used in this study to control gender variables. We recognize that the estrogenic environment may have specific regulatory effects on uric acid and bile acid metabolism. This is both a characteristic of the model and implies that the research conclusions need to be generalized to male individuals with caution. Future comparative studies between the sexes will be of great value to reveal possible gender dimorphism in hyperuricemia.

In conclusion, the present study demonstrated the alterations of bile acids and gut microbiota in the HUA rat model, particularly the CA synthesis in the liver and the levels of 12α-hydroxy BA in ileum contents. The correlations between ileum 12α-hydroxy BA and intestinal microbiota offered important clues to exploring the causes of increased 12α-hydroxy BA in the ileum. These findings allowed us to realize the roles of bile acids and gut microbiota in the HUA, providing a novel view for understanding the metabolic diseases among HUA patients.

## Data Availability

The data presented in this study are publicly available. This data can be found here: https://www.ncbi.nlm.nih.gov, accession number PRJNA134809.
